# Endoscopic management of a nail-magnet aggregation impacted in the terminal ileum: a case report

**DOI:** 10.1093/jscr/rjab259

**Published:** 2021-06-29

**Authors:** Matthew G K Benesch, Carrie L Howard, Chris G Smith

**Affiliations:** Discipline of Surgery, Faculty of Medicine, Memorial University of Newfoundland, St. John’s, NL, Canada; Discipline of Surgery, Faculty of Medicine, Memorial University of Newfoundland, St. John’s, NL, Canada; Discipline of Surgery, Faculty of Medicine, Memorial University of Newfoundland, St. John’s, NL, Canada

## Abstract

Management of inedible foreign objects within the gastrointestinal tract requires diligent observation and timely intervention in situations of impaction, obstruction or perforation. Here, we describe an adult patient with borderline personality disorder presenting with sequential ingestions of nails and magnets that had already passed beyond the reach of upper endoscopy. These objects conglomerated as a single mass in the terminal ileum and failed to clear into the colon despite a prolonged trial of conservative management. The patient underwent a successful colonoscopy to remove a significant component of the bezoar, allowing the remaining objects to pass through the ileocecal valve and be eliminated. This case highlights the benefits of careful conservative management with prudent use of endoscopy to manage impaction of foreign bodies in the terminal ileum in an otherwise stable adult patient.

## INTRODUCTION

Inedible foreign body ingestion is a management concern for general surgeons and gastroenterologists particularly in the pediatric population, incarcerated individuals and patients with psychiatric disorders [1]. Certain objects pose unique concerns. For example, button cells may cause thermal tissue destruction at the site of impaction, multiple magnets may attract each other though adjacent bowel wall layers and sharp objects may become embedded in the bowel wall [2]. The ultimate risk for any of these situations is bowel perforation which would require surgical management as sepsis can develop within a few hours of presentation [2, 3]. Prolonged impaction in the esophagus causing tissue ulceration and necrosis may form aorta-esophageal or trachea-esophageal fistulas [4].

In the absence of an acute complication such as obstruction or perforation, most inedible foreign bodies can be managed conservatively, especially if they are small and blunt (<2.5 cm in diameter and <5 cm in length) [2, 5]. These patients should be followed radiographically until elimination is confirmed [6]. Urgent upper endoscopy within 24 h is indicated for magnets, sharp or long objects >5 cm in length and within reach, and within 72 h for any object in the stomach unlikely to pass through the pylorus spontaneously [2, 5, 6].

In this case report, we describe a patient with sequential ingestion of nails and magnets that eventually impacted as a bezoar conglomeration in the terminal ileum. After these objects failed to move into the colon, the patient was successfully treated with colonoscopy to remove the bulk of the nails, allowing the remaining items to pass through the ileocecal valve.

## CASE REPORT

**Figure 1:**
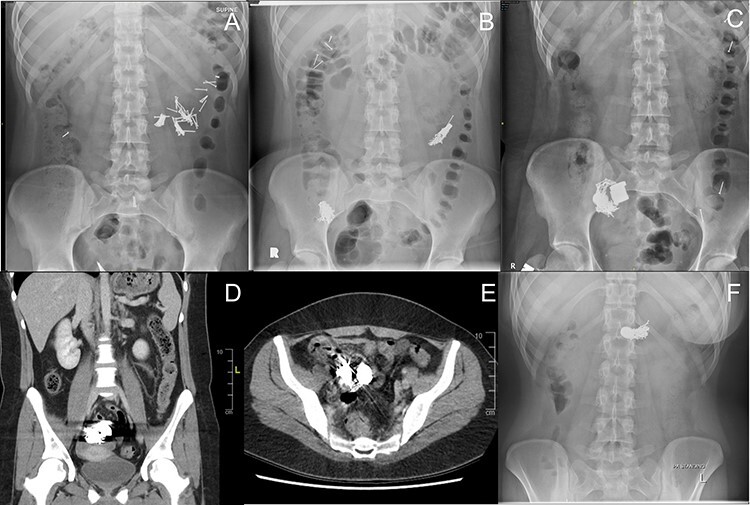
Radiographic series of sequentially ingested nails and magnets. The nails measured 2 cm long and magnets 2 cm in diameter and 0.75 cm in height. All dates are relative to initial ingestion of nails. (**A**) Day 1 – Nails in small bowel. (**B**) Day 6 – Additional ingestion of nails and a magnet. Original nails from A are clustered in right lower quadrant. (**C**) Day 9 – An additional four magnets have migrated to the right lower quadrant collection. (**D**) Day 15 – Coronal computer tomography (CT) view of nail-magnet collection in terminal ileum. (**E**) Day 15 – Axial CT view of nail-magnet collection in terminal ileum. (**F**) Day 26 – Smaller nail-magnet collection in transverse colon, 4 days after colonoscopic removal of the bulk of the collection.

A 20-year-old female with a long history of complex borderline personality disorder, post-traumatic stress disorder and factitious disorder characterized by multiple admissions for overdoses and foreign body ingestions presented to the emergency room following ingestion of multiple 2-cm finishing nails, dispersed throughout the small bowel on abdominal X-ray (Fig. 1A). She was admitted for observation but on the fourth day left against medical advice (AMA) and returned after swallowing additional nails and one magnet. This magnet measured radiographically with a dimeter of 2 cm and height of 0.75 cm. She was taken for an esophagogastroduodenoscopy using a pediatric gastroscope but the magnet had already passed beyond the proximal jejunum (Fig. 1B). Three days later she left AMA for the second time and returned having overdosed on acetaminophen and swallowed additional magnets. Two days afterwards, following medical clearance by internal medicine, the X-ray in Fig. 1C was taken showing an additional four magnets aggregated with nails and the first magnet in the right lower quadrant. Serial X-rays demonstrated no movement of this complex. She remained stable with intermittent abdominal pain but no clinical or radiographical signs of bowel obstruction. Six days later, a computer tomography scan of the abdomen showed this complex to be lodged within the terminal ileum (Fig. 1D–E).

**Figure 2:**
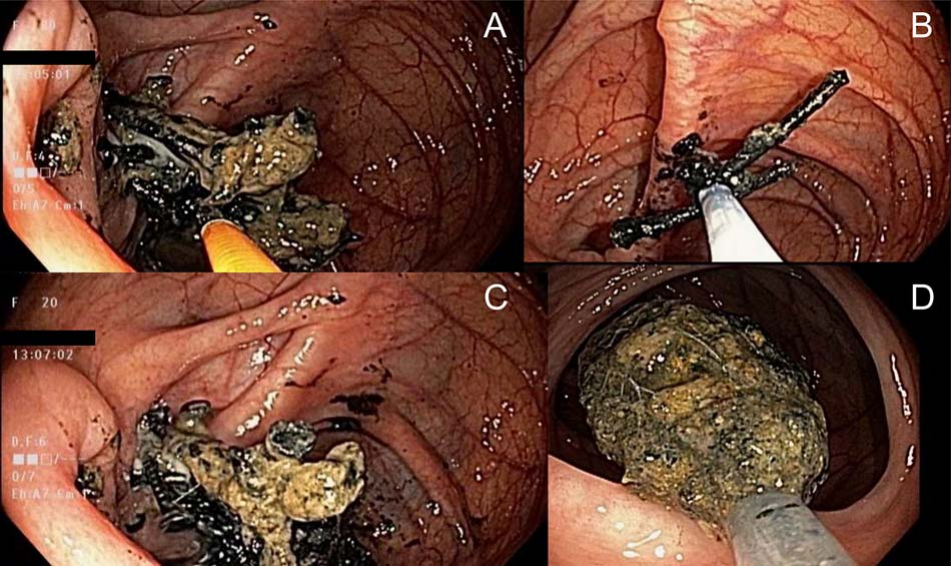
Endoscopic photographs of removal of nail-magnet collection from the terminal ileum. (**A**) Endoscopic forceps used to remove nails visible from the ileocecal valve (left of photograph). (**B**) Use of an endoscopic snare to remove additional nails from the ileocecal valve. (**C**) Collection of nails in cecum after their removal from the ileocecal valve. (**D**) Removal of the retrieved nails using a Roth Net® retrieval basket on colonoscopy withdrawal.

After another 8 days without movement the patient was prepared for a colonoscopy. A large conglomerate of nails was extracted from the bowel wall of the ileocecal valve with endoscopic forceps and snare (Fig. 2A–C) and placed in the cecum (Fig. 2C). Some of these nails appeared to be extracted through the wall of the cecum, raising the possibly of an enteric-colonic fistula formed by impaction of the mass. Intubation of the terminal ileum did not show any retrievable objects. A Roth Net® retrieval basket was used to extract a total of 10 collected nails from the cecum (Fig. 2D). Subsequent X-rays over the next several days showed migration of the remaining objects in colon (Fig. 1F) which were subsequently eliminated.

Unfortunately, despite multiple psychiatric interventions, this patient continued overdosing and ingesting foreign bodies, which would ultimately lead to her death 2.5 years later.

## DISCUSSION

Most endoscopic literature involving ingested inedible foreign objects relate to upper gastroscopy for removal of large, sharp or ulcerating objects that are pre-pyloric [7, 8]. One report describes colonoscopy in children for a button cell and safety pin removal from the terminal ileum [9]. Objects >2–2.5 cm in diameter tend not to pass through the pylorus or ileocecal valve, and objects longer than 5–6 cm are restricted by the duodenal sweep [5]. Conventional experience therefore dictates that objects that can pass beyond the ligament of Treitz should pass on their own [8]. These patients undergoing expectant management should have periodic radiographic imaging to ensure passage [6].

In this case report, the patient sequentially swallowed nails and magnets that individually passed through the pylorus and would likewise have passed through the ileocecal valve. However, their eventual collection proximal to the ileocecal valve created a mass too large to pass through. As this patient was in no serious distress and clinically stable, she was monitored for 2 weeks anticipating that bowel peristalsis might enable passage of this mass. This additional waiting time was deliberate as the patient had no previous surgical history and there were concerns operative intervention would exacerbate her underlying psychiatric comorbidities [10]. This suspicion was ultimately confirmed, as the patient would present numerous times in subsequent encounters over 2.5 years requiring multiple endoscopic and surgical procedures for complications by foreign objects. When conservative management failed, colonoscopic removal of a sufficient quantity of nails reduced the remaining mass to a sufficient size to pass spontaneously. We suggest that clinicians faced with a similar situation retrieve only what material is easily accessible to minimize the possibility of bowel trauma. Items retrieved into the cecum can be elected to pass on their own rather than endoscopically retrieving them. These patients should then be assessed by other allied health professionals as appropriate.
